# The Difficulties of Organising the Profession

**Published:** 1896-11-14

**Authors:** 


### THE DIFFICULTIES OF ORGANISING THE PROFESSION.

The continuance of complaints as to hospital abuse and as to what, in the elegant phraseology of the day, is called the "sweating" of the doctors, notwithstanding the endless schemes which are put forward for organising the medical profession into one solid phalanx strong enough to resist all aggressors and wipe away all abuses, must make thoughtful men inquire what is the cause of the comparative failure of all these efforts at reform. Plan after plan has been proposed, and time after time has the profession been rallied to arms to resist some onslaught on its prerogatives. But the result has been far from great, and in only a few places has any real triumph been obtained.

In regard to the so-called "battle of the clubs," it has long been clear that wherever a victory has been scored against the doctors, it has been the fault of weak members of the profession who have undersold their brethren; and as to hospital abuse, even the *Lancet* now admits—what we have all along maintained—that the medical staff are responsible for its continuance, and that its prevention lies in their hands. Yet they make no move! In view of these facts we may well ask, What is there in medicine which makes its practitioners so unorganisable; and why should doctors refuse to stand shoulder to shoulder for the common good, and to submit to sacrifices for the benefit of their brethren, with the same spirit and unanimity as is shown by miners and weavers and other common folk?

The answer, we think, is not far to seek. It lies in the intense individualism of medical work, and in the purely individual and personal character of medical success. The attractiveness of any business or profession depends far more on the prizes which it offers to the lucky few than on the certainty which it holds out to the laborious many. No one can pretend that the sort of men who become clergymen, doctors, and barristers are tempted by the meagre livelihood which these professions offer as a certainty to all who enter them. The prizes are the attractions, and for the sake of winning them men are willing to put up for a time with great privations—and in so doing they lower the average "wages" of their professional brethren.

This is especially true in regard to medicine. The normal reward for the hard work and long hours of a general practitioner would seem to be a little house, a sorry nag, an anxious wife, and about three hundred a year on which to "keep up appearances." But this is not the ideal that draws the students. They are attracted by what they hear of the great men, the dwellers in Harley Street and in Mayfair, the friends and confidants of princes and ministers of state. These are their guiding stars, and although they may know that no such luck can come to them, individualism is the keynote to all their actions, and personal individual success is what they aim at.

All this is destructive to union. The plodders may be glad enough to join anything which offers a certain income, but the young men who enter with great hopes look on their present troubles as a passing stage in their existence, a step to something better. They had rather bear the fate, spending long hours attending club patients or working for nothing at the hospital, than lessen by one iota their chance of success as practitioners or consultants, as the case may be.

It is universally recognised that the young men are the great difficulty in organising the profession. They care not how hard they work, they care not how low their fees, so that they can but see patients and get their chance of rising to something better. Of such men black sheep are made, and, no doubt, by such men the average reward of professional work is kept down. Nevertheless, such men and their ambitions must be reckoned with in any attempt to organise the profession, for among them may be found the best men of their years. The charming sketch of Sir James Simpson's life, given in this month's *Practitioner*, under the title of "From Baker's Boy to Baronet," shows well how grand a career medicine offers to men of genius; but the picture there given of his daily life, of his boundless energy, and of his unsparing expenditure of self in the furtherance of his work, also shows to those who care to read between the lines how intensely individual was all his work, and how difficult it must always be to "organise" a profession which contains such men. Fancy Sir James Simpson being tied down to a tariff as to fees!

The point, then, that must be borne in mind by those who would reform the medical profession is that experience derived from "unions," and from strikes and organisations of such people as weavers and miners, of trades in which the wages obtained are practically the same to all, and in which any advance of wage is shared equally by all, is totally inapplicable to such a profession as that of medicine, in which the recompense received by the successful bears no relation whatever to that obtained by ordinary "hands."

